# Possible Role of Circulating Bone Marrow Mesenchymal Progenitors in Modulating Inflammation and Promoting Wound Repair

**DOI:** 10.3390/ijms23010078

**Published:** 2021-12-22

**Authors:** Laura Grech, Jean-Paul Ebejer, Oriana Mazzitelli, Kevin Schembri, Joseph Borg, Elisa Seria

**Affiliations:** 1 Centre for Molecular Medicine and Biobanking, University of Malta, MSD 2080 Msida, Malta; jean.p.ebejer@um.edu.mt (J.-P.E.); oriana.mazzitelli@um.edu.mt (O.M.); 2 Department of Applied Biomedical Science, Faculty of Health Sciences, University of Malta, MSD 2080 Msida, Malta; joseph.j.borg@um.edu.mt; 3 Department of Surgery, Faculty of Medicine and Surgery, University of Malta Medical School and Mater Dei Hospital, MSD 2080 Msida, Malta; kevin.schembri@gov.mt

**Keywords:** bone marrow mesenchymal progenitor cells, wound healing, inflammation

## Abstract

Circulating bone marrow mesenchymal progenitors (BMMPs) are known to be potent antigen-presenting cells that migrate to damaged tissue to secrete cytokines and growth factors. An altered or dysregulated inflammatory cascade leads to a poor healing outcome. A skin model developed in our previous study was used to observe the immuno-modulatory properties of circulating BMMP cells in inflammatory chronic wounds in a scenario of low skin perfusion. BMMPs were analysed exclusively and in conjunction with recombinant tumour necrosis factor alpha (TNFα) and recombinant hepatocyte growth factor (HGF) supplementation. We analysed the expression levels of interleukin-8 (*IL-8*) and ecto-5′-nucleotidase (*CD73*), together with protein levels for IL-8, stem cell factor (SCF), and fibroblast growth factor 1 (FGF-1). The successfully isolated BMMPs were positive for both hemopoietic and mesenchymal markers and showed the ability to differentiate into adipocytes, chondrocytes, and osteocytes. Significant differences were found in *IL-8* and *CD73* expressions and IL-8 and SCF concentrations, for all conditions studied over the three time points taken into consideration. Our data suggests that BMMPs may modulate the inflammatory response by regulating *IL-8* and *CD73* and influencing IL-8 and SCF protein secretions. In conclusion, we suggest that BMMPs play a role in wound repair and that their induced application might be suitable for scenarios with a low skin perfusion.

## 1. Introduction

Wounds and tissue damage caused by trauma, inflammation, and infection can disrupt the normal cellular architecture of the tissue. In response to this, a repair process is triggered involving skin-resident and immune cells to restore tissue integrity through a series of regulated events.

Circulating bone marrow mesenchymal progenitors (BMMPs) are peripheral blood cells with a fibroblast-like shape that are able to migrate to regions of tissue injury [1]. These cells exhibit mixed morphological characteristics and express both hematopoietic stem cell antigens and markers of mesenchymal stem cells (MSCs) [2,3]. Studies have shown that BMMPs at sites of tissue damage favour tissue repair by acquiring antigen-presenting cells’ (APCs) properties. They also have secretion properties by producing cytokines, growth and angiogenic factors, and essential extracellular matrix (ECM) proteins [4,5,6]. BMMPs also secrete chemokines which recruit various types of immune cells and act in an autocrine manner leading to the migration of other stem cells to the site of tissue damage. Once they reach the wound site, BMMPs differentiate into secreting myofibroblasts and produce collagenous proteins and other ECM components [7,8,9], and promote scar formation [10].

Studies have also shown that mature MSCs play an important role in the immune system since they possess both anti-inflammatory and pro-inflammatory properties. They aid in limiting secondary damage by promoting inflammation when the immune system is under-activated and immunosuppression when it is over-activated [11,12].

Mature MSCs modulate immunosuppression by secreting indoleamine 2,3-dioxygenase (IDO), prostaglandin E2 (PGE2), and nitric oxide (NO). This process is triggered by pro-inflammatory cytokines such as tumour necrosis factor alpha (TNFα) and interferon-gamma (IFN-γ). They also secrete chemokines such as metalloproteinase-1 (MMP-1) and interleukin 8 (IL-8) which enhance the T-cell response, thereby exhibiting a pro-inflammatory phenotype [13,14,15].

MSCs’ immunomodulatory properties act on keratinocytes and dermal fibroblasts [16] and are therefore considered as a potential therapeutic alternative for the healing of skin defects including leg ulcers, trauma, burn wounds, and scar excision [11]. Favourable wound healing relies on the resolution of inflammation, which if altered or dysregulated might lead to poor healing outcomes.

This study aimed to determine whether an inflammatory state can trigger BMMPs to acquire their immunomodulatory and tissue remodelling properties. To achieve this, we used the skin model developed in our previous study [17] to study the behaviour and the possible effect of BMMPs in a typical chronic wound in patients with low skin perfusion and hence low leukocyte infiltration. Recombinant TNFα, as pleiotropic cytokine with a pro-inflammatory effect [18], and recombinant hepatocyte growth factor (HGF), as a paracrine cellular growth factor [19], were supplemented in order to create an inflammatory and a pro-regenerative state, respectively. The effect of the BMMPs was measured through the expression level of the genes encoding *IL-8* and ecto-5′-nucleotidase (*CD73*). IL-8, stem cell factor (SCF), and fibroblast growth factor (FGF-1) were measured at a protein level to evaluate the BMMPs’ effect after wound infliction and supplementation with TNFα and HGF.

## 2. Results

### 2.1. Isolation, Culture, and Characterisation of Bone Marrow Mesenchymal Progenitor Cells

**Figure 1:**
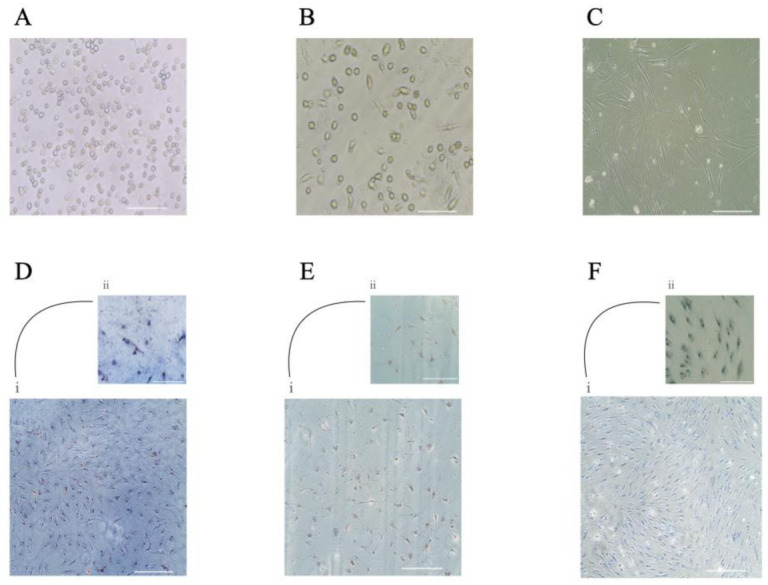
Isolation and culture of circulating BMMPs. Freshly isolated Buffy-coat (**A**), adherent BMMPs showed their characteristics to form colonies (**B**), and their spindle-shape figure (**C**). Photos refer to day 0 (**A**) and to day 4 (**B**) and day 15 (**C**) of the primary culture. Scale bar = 50 μm (**A**–**C**). BMMPs showed an ability to differentiate into adipocytes (**D**i,ii), osteocytes (**E**i,ii), and chondrocytes (**F**i,ii). Scale bar= 100 μm (**D**i**E**i**F**i); Scale bar = 50 μm (**D**ii,**E**ii,**F**ii).

**Figure 2:**
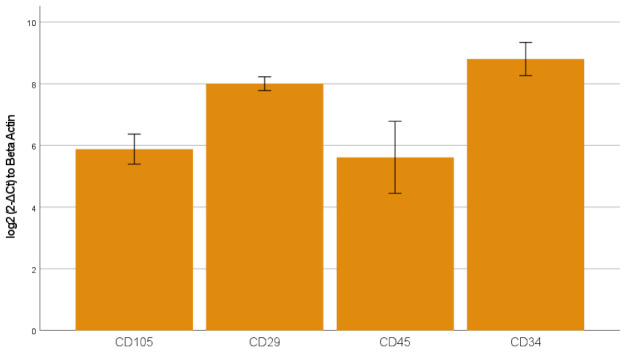
mRNA expression of genes involved in mesenchymal and haematopoietic lineages was determined by qPCR. Transcript levels were normalized to the ACTB reference gene using log2 (2-ΔCt) method. The data is presented as mean  ±  standard error (SE). The graph bar shows expression level of the genes CD105, CD29, CD45, and CD34.

The freshly isolated and seeded peripheral blood-derived mononuclear cells (PBMCs) showed a small and round morphology (Figure 1A). After 48 h, the non-adherent cells were removed and from day four, the adherent mesenchymal progenitor cells showed their ability to form colonies (Figure 1B) and exhibited their typical BMMP spindle-shape morphology (Figure 1B,C). Gene expression analysis showed the presence of specific markers for mesenchymal (CD105 (6 ± 0.7), CD29 (8.1 ± 0.24)) and haematopoietic (CD45 (5.6 ± 1.4), CD34 (8.8 ± 0.6)) lineages (Figure 2).

The ability of BMMPs to differentiate into the three lineages was observed both microscopically and through histological staining. Adipocytic differentiation was completed after 14 days. This was confirmed by Oil Red O staining which showed the presence of characteristic lipid vacuoles in the cytoplasm (Figure 1D). Osteogenic and chondrogenic differentiation was reached after 21 days as determined by Von Kossa for dark-coloured calcium deposits and by Alcian blue for proteoglycans staining, respectively (Figure 1E,F).

**Figure 3:**
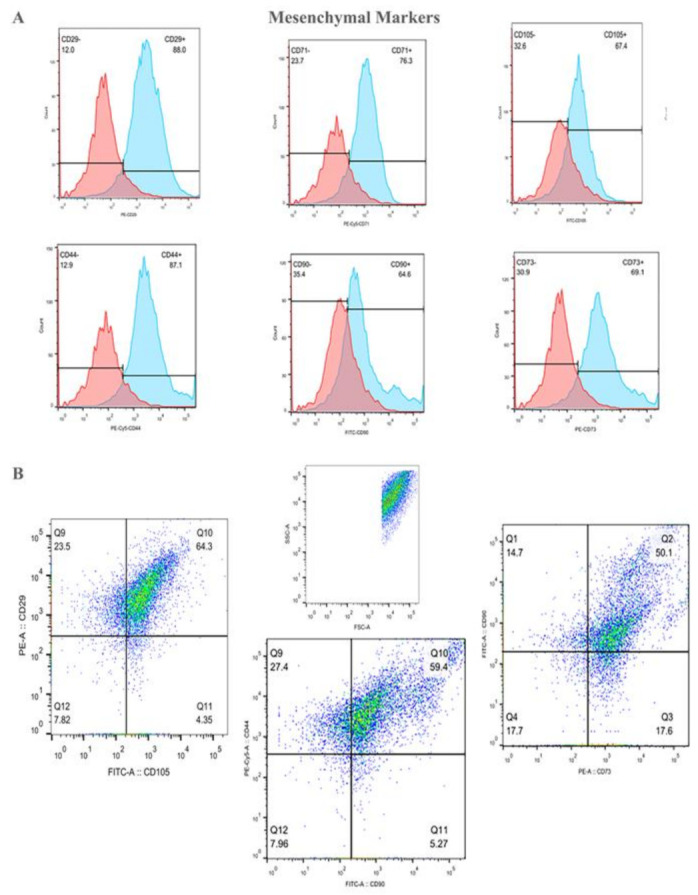

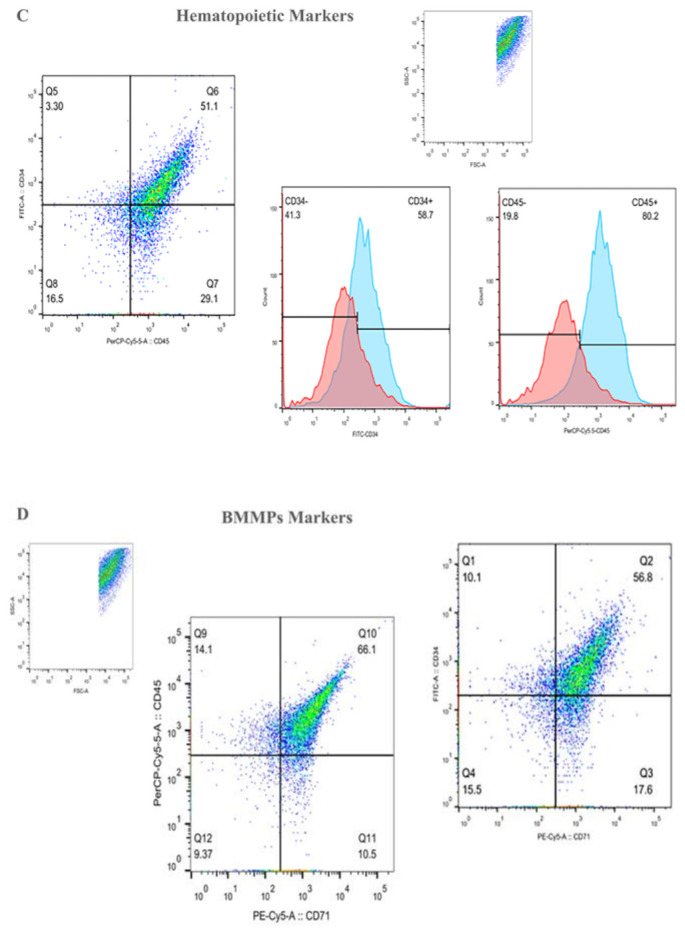
The population of the adherent Bone Marrow Mesenchymal Progenitors (BMMPs) was acquired and analyzed using the Forward scatter (FSC) and the Side scatter (SSC) distribution of the cells (**B**–**D**). Histogram plots show the positivity for the mesenchymal markers CD29, CD71, CD105, CD44, CD90, and CD73 (**A**), with a high positive percentage for double stained population CD29/CD105, CD90/CD44 and CD90/CD73 (**B**). BMMPs were also highly positive for the hematopoietic markers CD34 and CD45 (**C**) alone and in co-expression with the CD71 MSCs marker (**D**).

The phenotypes of the BMMPs displayed mesenchymal markers CD29 (83.6% ± 4.7), CD71 (74% ± 2.1), CD105 (62.5% ± 5.3), and CD44 (85% ± 1.9), CD90 (65.5% ± 5.8), CD73 (69.6% ± 4.0) (example dot plot, Figure 3A), with a high positive percentage for double stained population CD29/CD105 (58.7% ± 6.5), CD90/CD44 (65.2% ± 4.5) CD90/CD73 (53.5% ± 8.4), (example dot plot, Figure 3B). They also highly expressed the hematopoietic markers CD34/CD45 (53.3% ± 2.4), CD34 (65% ± 5.6), and CD45 (76.9% ± 2.9) (example dot plot, Figure 3C), and interestingly displayed a double positive population for the hematopoietic markers CD45 and CD34, and the CD71 MSCs marker [20] (CD45/CD71 63.10% ± 3.0; CD34/CD71 55.27% ± 3.2) (example dot plot, Figure 3D). At first passage and prior to the infusion to the wound model, the viability of the BMMPs was 85% in culture.

### 2.2. Effects of Scratch Injury Assay, and Supplementation of Tnfα, HGF, and BMMPs on Gene Expression in the Skin Equivalent Model

We investigated the response of skin-resident cells on our model in relationship with eight different experimental conditions at three different time points using an unscratched control as a reference.

**Figure 4:**
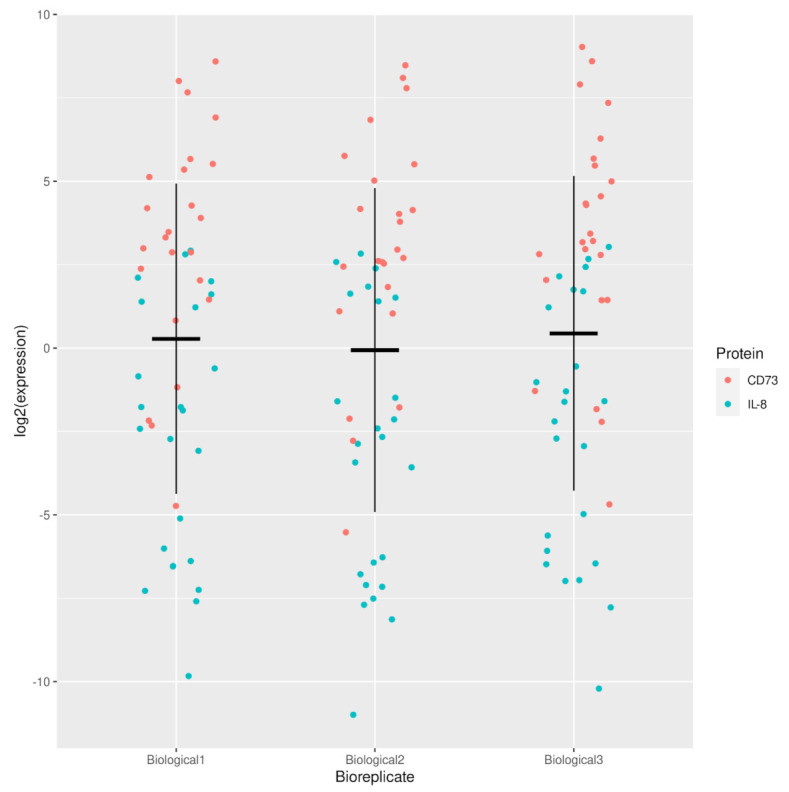
Strip plot showing the log2 expression of IL-8 and CD73 for the three biological replicates for all conditions.

**Figure A1:**
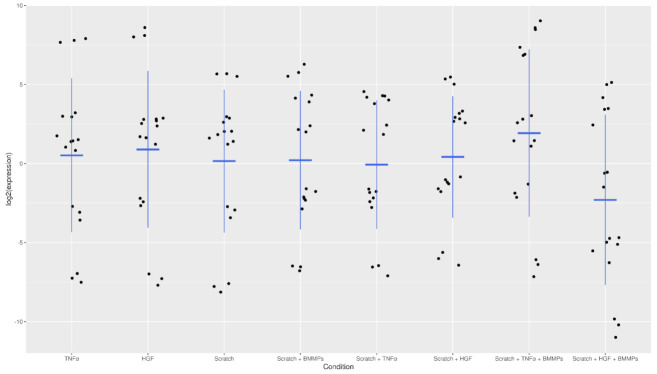
Strip plot of log2 expression of IL-8 and CD73 against the different conditions for the three biological replicates.

For this purpose, qPCR was performed to evaluate the expression levels of *IL-8* and *CD73*, two genes known to be involved in the inflammatory process of wound healing. Overall, low variation was observed between the three biological replicates, for all nine different conditions and different time points (Figure 4 and Appendix A Figure A1) used in the experiment.

**Figure 5:**
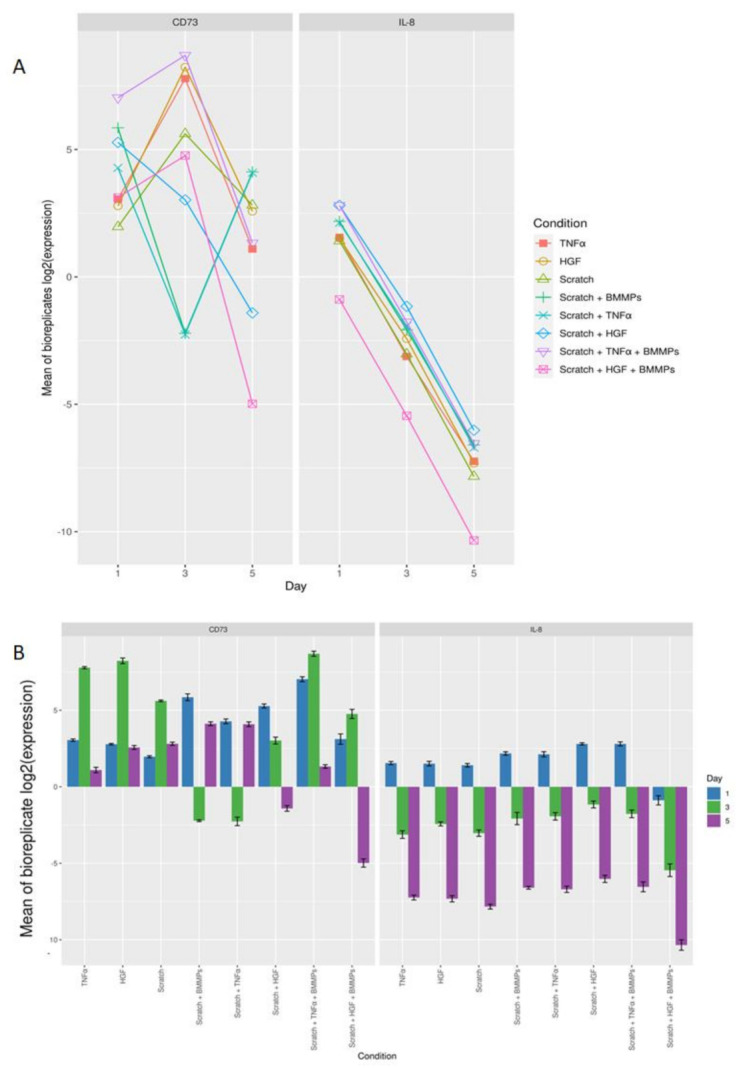
(**A**): Log2 expression for the mean of biological replicates for *IL-8* and *CD73* for all conditions at the three different time points. (**B**) Bar graph showing the log 2 expression mean of the biological replicates of *CD73* and *IL-8* for the eight different conditions on day one, day three, and day five.

**Figure A2:**
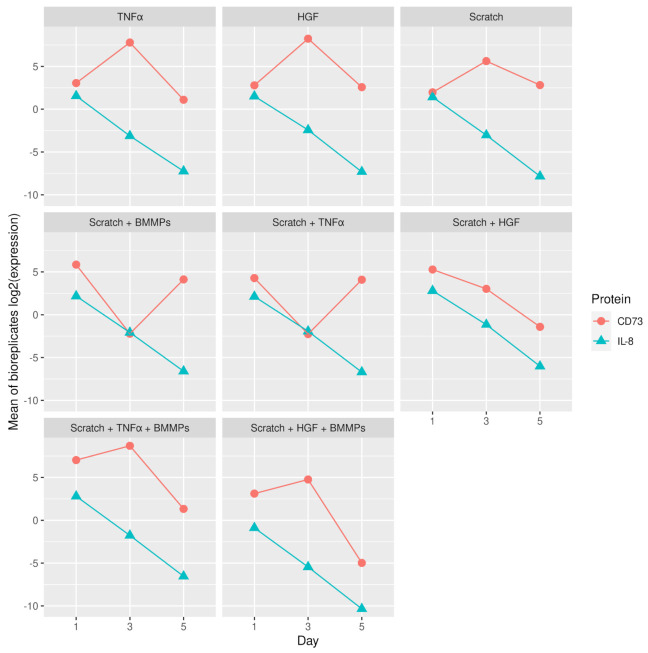
Log2 expression of the mean of three biological replicates for IL-8 and CD73 for the three different time points.

A progressive decrease in *IL-8* expression was noted in all conditions from day one to day five (Figure 5A,B and Appendix A Figure A2). The most prominent significant difference in *IL-8* expression was seen on day three between scratch + HGF + BMMPs compared to scratch + BMMPs and scratch + HGF + BMMPs compared to the supplementation of HGF alone (*p* = 1.14417 × 10^−11^ and *p* = 5.00767 × 10^−10^) (Table 1). The adjusted *p*-values for all conditions and interactions between conditions and days is shown in Supplementary Materials).

With regards to *CD73* expression, overall, the most statistically significant difference was noted between scratch + BMMPs when compared to TNFα supplementation alone (*p* = 8.135 × 10^−12^), scratch + HGF + BMMPs when compared to scratch + HGF (*p* = 2.382 × 10^−11^), and scratch + HGF + BMMPs when compared to scratch + HGF (*p* = 1.095 × 10^−10^) (Table 1 and Appendix A Figure A2). In terms of days, the most statistically significant difference in *CD73* expression was noticed on day five with scratch + TNFα + BMMPs when compared to scratch + TNFα (*p* = 4.796 × 10^−14^). An upregulation of *CD73* was noted after the infliction of the wound and even in relation to the TNFα supplementation. On the other hand, *CD73* was downregulated after the infusion of the wound in combination with TNFα supplementation. The infusion of the BMMPs led to an upregulation of *CD73* (Figure 5A,B and Figure A2). The HGF supplementation promoted *CD73* upregulation, which was inhibited by the infliction of the wound plus the supplementation of HGF, while slightly augmented by the BMMPs’ infusion. Interestingly, the infusion of BMMPs in the wound site resulted in the downregulation of *CD73* as happened in the wounded condition plus the supplementation of TNFα and HGF.

### 2.3. Protein Secretion

**Figure 6:**
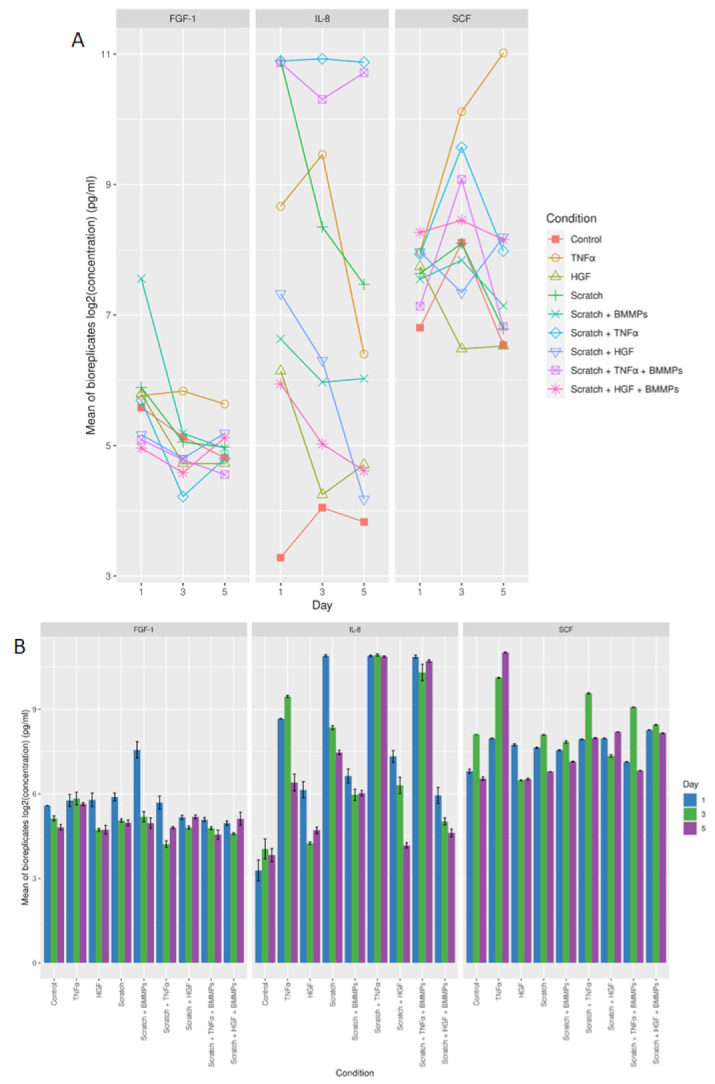
(**A**) Log2 concentration in pg/mL (mean of biological replicates) for FGF-1, IL-8, and SCF protein for all the different conditions and time points. (**B**) Bar graph showing the log2 mean of biological replicates for FGF-1, IL-8, and SCF protein for all the different conditions on day one, day three, and day five.

**Figure 7:**
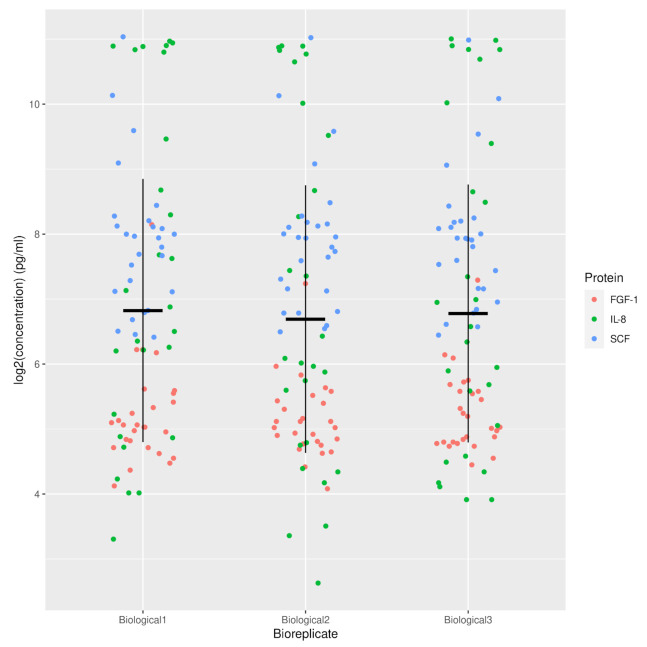
Strip plot of log2 concentration in pg/mL against the different biological replicates for secretion of FGF-1, IL-8, and SCF proteins.

**Figure A3:**
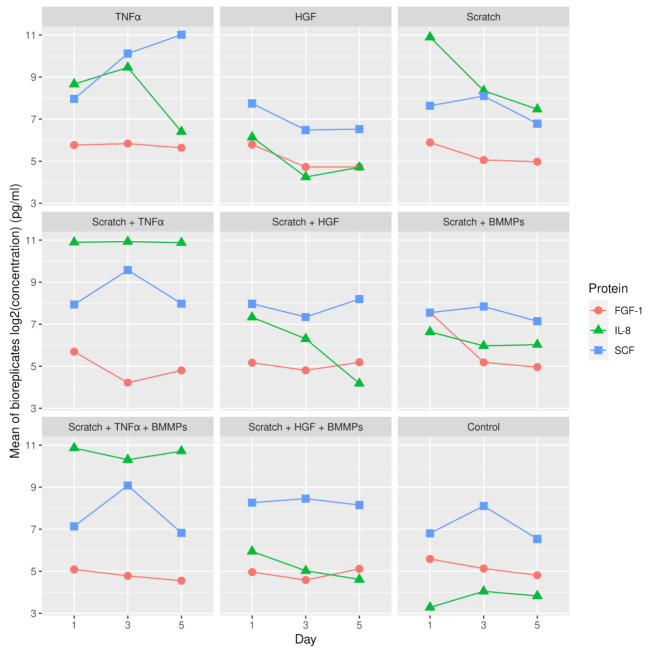
Log2 concentration in pg/mL of mean of biological replicates for FGF-1, IL-8, and SCF protein for the three different time points.

We investigated the secretion of IL-8, SCF, and FGF-1 in the cell culture medium of the nine skin model conditions studied. These were measured at several time points by ELISA assay. At the protein level, different protein secretion was noted between all conditions for the three time points taken into consideration (Figure 6A,B and Appendix A Figure A3) with low inter-variability between the three biological replicates (Figure 7). A significant difference was noted in IL-8 secretion between the scratch + TNFα + BMMPs condition and the control condition on day one and three after wounding (*p* = 4.37 × 10^−13^ and *p* = 4.37 × 10^−13^ respectively). Conversely, on day one after wounding, the scratch + HGF + BMMPs condition showed a significantly lower IL-8 secretion (*p* = 4.37E-13). Interestingly, on day three and on day five after wounding, the scratch + HGF + BMMPs condition showed lower IL-8 secretion when compared with the HGF condition and the scratch + BMMPs condition (*p* = 0.003 and *p* = 0.000 respectively). These observations could suggest a joint effect of HGF supplementation and BMMPs infusion in inhibiting IL-8 secretion.

On day one after wounding, the BMMPs infusion led to a significant increase in FGF-1 secretion compared to the scratch + HGF + BMMPs and the scratch + TNFα + BMMPs conditions (*p* = 4.67 × 10^−13^ and *p* = 5.18 × 10^−13^). The TNFα supplementation led to a significantly higher FGF-1 secretion compared to the scratch + TNFα + BMMPs for both day three and day five post-wound (*p* = 0.001 and *p* = 0.000). With regards to SCF production, a statistically significant difference was noted after the infusion of the BMMPs alone and the concomitance of HGF supplementation after wounding. This was specifically noted on day three when comparing the scratch + HGF + BMMPs condition with scratch + HGF condition (*p* = 4.37 × 10^−13^) and the scratch + HGF + BMMPs condition with the HGF condition (*p* = 4.37 × 10^−13^) (Table 2).

## 3. Discussion

In this study, we isolated circulating BMMPs focused mainly on their cellular and molecular role in wound healing. Our previously published skin wound model [17] was used to observe the immune regulatory effect of the BMMPs during the inflammatory phase of wound healing. The gene expression levels of *IL-8* and *CD73* and of the soluble proteins IL-8, FGF-1, and SCF were measured in nine different experimental conditions at three different time points.

The inflammatory phase of wound healing is characterised by the invasion of phagocytic neutrophils in the damaged area in the first 24 h after injury, which can last for several days [21]. IL-8 is one of the most neutrophil chemoattractants produced during the inflammatory cascade [22]. Apart from IL-8, the pro-inflammatory cytokine, TNFα is released by keratinocytes and fibroblasts in the injured area. An increase in the production of TNFα also occurs in the first 12–24 h after injury. This indicates that TNFα is closely involved in the initial process of wound healing in skin [23,24]. In this study, when compared to the control, *IL-8* expression was upregulated in all experimental conditions on day one after the scratch injury. This was also noted at the protein level, thereby confirming the induction of the inflammatory phase after wounding. *IL-8* was downregulated from day three onwards in all conditions.

TNFα controls the activity of fibroblasts, inducing proteoglycans and fibronectin production, and the secretion of the cytokines such as IL-1, IL-2, IL-6, and IL-8 [25,26]. In this study, the supplementation of TNFα showed a difference in SCF secretion when compared with FGF-1 and IL-8. SCF was secreted at higher levels on all the three time points when compared to the other conditions (Figure 6A,B). On the other hand, the infliction of the wound and subsequent supplementation of TNFα resulted in higher IL-8 secretion when compared to FGF-1 and SCF for all three time points studied. The infliction of the wound and the subsequent TNFα supplementation and infusion of BMMPs also led to a significant difference in IL-8 secretion when compared to FGF-1 and SCF.

Skin-resident cells produce a variety of growth factors in response to damage such as vascular endothelial growth factor (VEGF), platelet-derived growth factor (PDGF), epidermal growth factor (EGF), transforming growth factor beta (TGF-β), and HGF [26,27,28,29]. During the inflammatory phase, growth factors such as TGF-β, PDGF, and IL-1 mediate increased capillary permeability, leukocyte migration, and macrophage activation into the injured tissue [30,31]. TGF-β also promotes re-epithelialization, angiogenesis and ECM deposition [32]. Previous studies have shown that during the wound healing process, two types of cells with different tissue origins appear to be involved in collagen and ECM molecule synthesis. These are the fibrocytes which are the circulating BMMPs and fibroblasts, which originate from mature tissues [33]. Circulating BMMPs interact functionally with the skin-resident cells to promote wound repair by secreting chemokines, cytokines, and angiogenic and fibrotic factors, such as EGF, basic fibroblast growth factor (bFGF), and PDGF [8,34,35]. The high secretion of TGF-β that occurs during the inflammatory and proliferation phase of wound healing could lead to the maturation of the circulating BMMPs into myofibroblasts and mature BMMPs [1].

Ecto-5′-nucleotidase is an immunosuppressive molecule which has both an anti-inflammatory and anti-fibrotic effect and contributes to the production of extracellular adenosine [36,37,38]. CD73 also maintains vascular endothelial integrity [39], which helps the development of bone marrow-derived mesenchymal stem cells as well as in providing protection from bone injuries [40]. A study conducted in an animal mice model by Shun et al. showed that CD73 mitigated the inflammation which occurs in a spinal cord injury by stimulating macrophages/microglia M2 polarization [41]. In our wound experiment, the *CD73* was upregulated on day one in five different experimental conditions, with a peak on day three, and a subsequent downregulation on day five, as a result of a normal inflammatory response of the skin resident cells.

**Figure 8:**
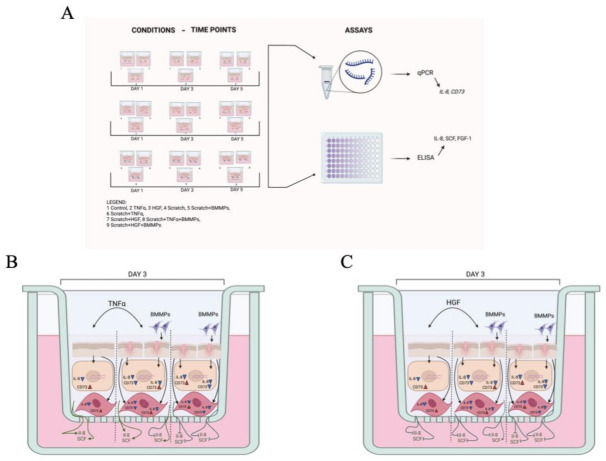
(**A**) Experimental wound model. Schematic representation of the nine experimental conditions: unscratched skin (control, TNFα, and HGF supplemented), scratched skin (scratched, TNFα, and HGF supplemented), and scratched skin and BMMPs infused (scratched, TNFα, and HGF supplemented, followed by BMMPs infusion). The scheme also shows the three time points taken into consideration (days one, three, and five after wound infliction). qPCR and ELISA were performed to measure expression levels of *IL-8* and *CD73* and protein levels of IL-8, SCF, and FGF-1, respectively (**B**). Schematic representation of the different experimental conditions on skin models on day three after wound infliction (**C**). The schemes show the effect of TNFα (**A**) and HGF (**B**) supplementation and BMMPs infusion with and in absence of damage. Evaluation of their effects was conducted at molecular level, by gene expression level quantification of *IL-8* and *CD73*, and at protein level measurement of IL-8 and SCF. Created with BioRender.com. https://biorender.com/ (Accessed on 15 November 2021).

Mature MSCs constitutively, or upon stimulation, secrete large amounts of cytokines and growth factors. Pro-inflammatory cytokines act as a trigger for MSCs to exert an immunosuppression by secreting elevated levels of soluble factors, including IL-10, IDO, PGE2, NO, HGF, haem oxygenase (HO), and cyclooxygenase-2 (COX-2) [42,43]. In addition, mature MSCs also secrete pro-inflammatory phenotype chemokines including MMP-1 and IL-8, which enhances the T-cell response [15,44]. This pro-inflammatory behaviour might explain the downregulation of *CD73* on day three, followed by an upregulation on day five in the scratch and BMMPs infused conditions in our model. Secreted growth factors such as granulocyte colony-stimulating factor (G-CSF), granulocyte macrophage colony-stimulating factor (GM-CSF), SCF, TGF-β, VEGF, EGF, FGF, and HGF have a protective effect and play a role in re-epithelialization, epidermal cell differentiation, fibrosis, and angiogenesis during wound healing [45,46]. HGF is expressed in keratocytes, epithelium, and endothelium, and promotes the proliferation and cell development of epithelial cells [47,48]. It was also shown that HGF promoted corneal epithelial wound healing leading to epithelial cell proliferation, and suppressed corneal inflammation [49]. Our data shows a pronounced difference between scratch + BMMPs + TNFα / HGF and other conditions on day three after wound infliction (Figure 8).

An increased SCF production was noted following HGF supplementation and BMMPs infusion, and a significant difference in *IL-8* and *CD73* expression was noted when compared with an identical condition which lacked BMMPs. The infusion of BMMPs to the wound model, together with the supplementation of TNFα produced a significant upregulation of *CD73* when compared with an identical condition which lacked BMMPs. At the protein level, while IL-8 was highly detected in the scratch + TNFα condition, the infusion of BMMPs reduced IL-8 secretion and accumulation in the cell culture medium.

## 4. Materials and Methods

This research was approved by University of Malta’s research code of practice and research ethics review procedures (UREC, ref. no 56120 L7).

### 4.1. Isolation, Culture and Viability Assay of Circluating Bone Marrow Mesenchymal Progenitors

Buffy-coat units were obtained from routine processing of whole blood donated by healthy volunteers at the National Blood Transfusion Center. Isolation of circulating BMMPs was done using a gradient density media separation on Histopaque-1077 (Sigma-Aldrich, Burlington, MA, USA) by centrifugation at 500× *g* for 25 min at 20 °C. The isolated mononuclear cells were washed with PBS, centrifuged at 100× *g* for 10 min at room temperature, and resuspended in 12 mL of fresh cell culture medium (MesenCult Proliferation Kit, Stem Cell Technologies, Cambridge, UK). Antibiotics and antifungal treatment were added to the medium according to the following working solution concentrations: 50 mg/L gentamicin, 2500 μg/L amphotericin B, and 5000 units/L penicillin and streptomycin (Sigma-Aldrich, Burlington, MA, USA). BMMPs were seeded in a 75 cm^2^ flask and cultured at 37 °C in a humidified atmosphere and 5% CO_2_. Non-adherent cells were removed after 48 h. Media were replaced every 3 to 4 days until confluence was reached. BMMPs were counted and their viability was assessed with Countess TM II Automated Cell Counter using a 0.4% Trypan Blue solution (Thermo Fisher Scientific, Waltham, MA, USA) as per the manufacturer’s protocol. The assays were performed in triplicate at the first passage of the culture and before the infusion into the experimental models.

### 4.2. Flow Cytometry Analysis of the Circulating Bone Marrow Mesenchymal Progenitors

Cytofluorimetric characterization of BMMPs was done using monoclonal antibodies (moAbs) against the surface antigens FITC-CD105 (Endoglin, clone 43A3, Bio Legend, Amsterdam, The Netherlands), PE-CD29 (Integrin beta-1, clone TS2/16, Bio Legend), PE-Cy5 CD44 (CD 44 molecule, clone IM7, eBioscience), PE-Cy5-CD71 ( Transferrin receptor, T9, clone RI7217, Bio Legend, Amsterdam, The Netherlands), PE-CD73 (Ecto-5′-nucleotidase, clone AD2, Bio Legend), FITC-CD90 (Thy1, clone 5E10, Bio Legend, Amsterdam, The Netherlands), PE-CD34 (CD34 molecule, clone 561, Bio Legend, Amsterdam, The Netherlands) and Peridinin Chlorophyll Protein Complex (PercP)-CD45 (Leukocyte common antigen, clone 2D1, Bio Legend, Amsterdam, The Netherlands). The unstained cell population was used as corresponding negative control. When the adherent BMMPs reached 70% confluency at 1st passage they were treated with 0.05% Trypsin-EDTA for 10 min at 37 °C. The addition of 10% FBS in PBS was used to stop the enzymatic reaction. Cells were washed with PBS and 200μL of cell suspension was stained and incubated for 20 min at room temperature in the dark with 10μL of monoclonal antibody (moAb). The cell suspension was analysed with FACS Aria III (FACS Diva version 6.1.2, Becton Dickinson, Oxford, UK) and FlowJoTM v10.8 (BD Biosciences, Oxford, UK) was used to analyse the raw data. Initial forward scatter (FSC) and side scatter (SSC) distribution parameters of the cell populations were applied to exclude cell debris. Reading was assessed in triplicate.

### 4.3. Differentiation of the BMMPS into Osteocytes, Chondrocytes, and Adipocytes

Differentiation of BMMPs into osteocytes, chondrocytes, and adipocytes was induced using Gibco StemPro™ Supplement kits (Thermo Fisher Scientific, Waltham, MA, USA) according to the manufacturer’s protocol. Each medium was augmented with antibiotics and anti-fugal treatment according the following working solution concentrations: 50 mg/L gentamicin, 2500 μg/L amphotericin B, and 5000 units/L/penicillin and streptomycin (Sigma-Aldrich, Burlington, MA, USA).

BMMPs differentiation was assessed using standard histological protocols. Chondrocytes were stained with Alcian Blue, adipocytes with Oil Red O and osteocytes with Von Kossa. Adipocytes were counterstained with Mayer’s Haematoxylin Solution whilst a 5% Van Gieson solution was used for osteocytes and chondrocytes.

### 4.4. The In Vitro Experimental Model of Wound Healing

The in vitro skin model was assembled in a 24-well plate using a 6.5 mm trans-well with a 0.4 μm pore polyester membrane insert and a cell growth area of 0.33 cm^2^ (Corning Trans-well, Sigma-Aldrich). In order to resemble a skin wound, a scratch injury of 0.5 cm length was inflicted using a 100 μL sterile tip. Supplementation of 100 ng/mL of human TNFα (Bachem AG, Switzerland) was used to mimic an inflammatory condition. Supplementation of 100 ng/mL of human HGF (Bachem AG, Switzerland) was used as a proliferative factor. Both growth factors were supplemented 3 h after the infliction of the wound. 6 × 10^4^cells/cm^2^ BMMPs, resuspended in 10 µL of normal saline solution (0.9%), were infused in simultaneity with TNFα and HGF supplementation.

In this study, nine different conditions of skin/wound model were created as follows: (i) Control: non-injured skin model used as control condition, (ii) TNFα: non-injured skin model followed by rTNFα supplementation, (iii) HGF: non-injured skin model followed by HGF supplementation, (iv) Scratched: a scratched skin model without the supplementation of either TNFα or HGF and mesenchymal progenitor cells infusion, (v) Scratched + BMMP: a scratched skin model followed by BMMP infusion, (vi) Scratched + TNFα: a scratched skin model followed by TNFα supplementation, (vii) Scratched + HGF: a scratched skin model followed by HGF supplementation, (viii) Scratched + TNFα + BMMP: a scratched skin model followed by TNFα supplementation and BMMPs infusion, and (ix) Scratched + HGF + BMMPs: a scratched skin model followed by HGF supplementation and BMMPS infusion.

Skin equivalent models were performed in three biological replicates and for each condition, all conditions were established in experiments at three time points (days 1, 3, and 5). At each time point, we conducted quantitative-PCR (qPCR) for *IL8* and *CD73* genes, I, and enzyme-linked immunosorbent assay (ELISA) for measurement of IL-8, SCF, and FGF-1 proteins. (Figure 8).

### 4.5. RNA Extraction and qPCR

The skin models were digested prior to RNA extraction. According to our previous published protocol [17] RNA was extracted from 5 × 10⁶ adherent circulating BMMPs and the skin model using the Pure Link^®^ RNA Mini Kit (Thermo Fisher Scientific, Waltham, MA, USA) as per manufacturer’s protocol.

RNA was measured and its quality verified using Nanodrop 2000 (Thermo Fisher Scientific, Waltham, MA, USA). A total of 50 ng RNA from each sample was used to produce a complementary DNA (cDNA) using Revert Aid™ First Strand cDNA Synthesis Kit (Thermo Fisher Scientific, Waltham, MA, USA). Three technical replicates of qPCR were performed using Rotor-Gene Q Series Software 2.1.0. (Qiagen, Hilden, Germany) as follows: 1 µL cDNA was added with 5 × HOT FIREPol Eva Green qPCR Super mix (Solis BioDyne, Tartu, Estonia), primers for CD34, CD45, CD29, CD105, CD73, IL-8, and ACTB (Table 3), and PCR grade water to formulate 20 µL reaction. Thermal cycling was done for 12 min at 95 °C followed by 40 cycles at 95 °C for 15 s, 60 °C for 30s, and 72 °C for 30 s. mRNA expressions were normalised against the endogenous reference gene ACTB through the comparative Ct method (2^(-∆∆Ct) and reported as fold change. Unscratched samples were used as calibrators.

### 4.6. Enzyme-Linked Immunosorbent Assay

Enzyme-linked immunosorbent assay (ELISA) (Thermo Fisher Scientific) was used to measure the secretion levels of IL-8, FGF-1, and SCF in culture medium and was performed in two technical replicates. The optical density (OD) was read using a multimode micro-plate reader (Mithras LB940) at an optical density of 450 nm. Measurements of unused culture medium were used as blanks and a curve-fitting standard curve was plotted for data analysis.

### 4.7. Statistical Analysis

R (version 4.1.2) was employed for the statistical analyses of the ELISA and qPCR data. In both cases the analysis followed a balanced design of the experiment. The alpha threshold of significance was taken at 0.05 throughout this study.

ELISA Data. The measured ELISA protein concentrations were transformed using logarithms (base 2). The time point (i.e., day) was not considered as a repeated measure, as the concentration at each time point was measured independently. Data was partitioned by protein (i.e., IL-8, SCF, FGF-1), and separate models were built for each protein. A two-way ANOVA was performed (using aov) to analyse the effect of condition, day, and their interaction on the (log2 of the) protein concentration. The two-way ANOVA revealed that there was a statistically significant interaction between the effects of condition and day for all three proteins. Simple main effects analysis showed that, individually, both condition and day have a statistically significant effect on the protein concentration. The test statistics for the three proteins are shown in Table 4. A Tukey post-hoc test was employed to identify which differences were significant. The full list of comparisons is available as Supplementary Table S1.

qPCR Data. The measured qPCR gene expression values were transformed using logarithms (base 2). The time point (i.e., day) was not considered as a repeated measure, as the expression at each time point was measured independently. Data was partitioned by gene (i.e., *CD73*, *IL-8*), and separate models were built for each gene. A two-way ANOVA was performed (using aov) to analyse the effect of condition, day, and their interaction on the (log2 of the) gene expression. The two-way ANOVA revealed that there was a statistically significant interaction between the effects of condition and day for *CD73* but not for *IL-8.* Simple main effects analysis showed that, individually, both condition and day have a statistically significant effect on the gene expression for both *CD73* and *IL-8*. The test statistics for the three genes are shown in Table 5. A Tukey post-hoc test was employed to identify which differences were significant. The full list of comparisons is available as Supplementary Materials.

## 5. Conclusions

Circular BMMPs might have a physiological role in tissue homoeostasis and repair. BMMPs may regulate the inflammatory cascade during the wound healing process by exerting their effect directly on skin-resident cells.

In this study we showed that peripheral blood (PB) can be considered as a possible source for these cells. Our previously developed leukocytes-depleted skin model was used to mimic a chronic wound occurring in patients with low skin perfusion, and subsequently to evaluate the immunomodulatory properties of BMMPs.

Our data suggests that infused circulating BMMPs might play a role in the modulation of the inflammatory response, specifically by anticipating the immunosuppressive response under inflammatory stimuli. This was shown by the anticipated upregulation of *CD73* and the inhibition of IL-8 secretion on day three in the condition supplemented with TNFα. This was also noted on day five for the condition supplemented with BMMPs only. BMMPs might therefore be used in a therapeutic approach for the treatment of chronic inflammatory wounds in a low skin perfusion scenario.

## Figures and Tables

**Table 1:** The most statistically significant conditions plus time points for IL-8 and CD73 expression.

Gene	Condition and Day	Adjusted *p*-Value	Gene	Condition and Day	Adjusted *p*-Value
IL-8	scratch + HGF + BMMPs:3-scratch + BMMPs:3	1.144 × 10^−11^	CD73	scratch + TNFα + BMMPs:5-scratch + TNFα:5	4.796 × 10^−14^
IL-8	scratch + HGF + BMMPs:3-HGF:3	5.007 × 10^−10^	CD73	scratch + TNFα + BMMPs:1-scratch + TNFα:1	2.999 × 10^−13^
IL-8	scratch + HGF + BMMPs:5-HGF:5	5.065 × 10^−10^	CD73	scratch + HGF + BMMPs:1-scratch + BMMPs:1	1.030 × 10^−12^
IL-8	scratch + HGF + BMMPs:5-scratch:5	1.177 × 10^−7^	CD73	scratch:3-HGF:3	1.505 × 10^−11^
IL-8	scratch + HGF + BMMPs:3-scratch:3	3.034 × 10^−7^	CD73	scratch + HGF:3-scratch:3	1.995 × 10^−11^
IL-8	scratch + HGF + BMMPs:1-HGF:1	3.849 × 10^−7^	CD73	scratch + HGF:1-HGF:1	8.371 × 10^−11^
IL-8	scratch + HGF + BMMPs:1-scratch:1	1.220 × 10^−6^	CD73	scratch + TNFα:1-scratch:1	9.267 × 10^−10^
IL-8	scratch + HGF:3-scratch:3	0.000	CD73	scratch + HGF + BMMPs:1-scratch + HGF:1	7.003 × 10^−9^
IL-8	scratch + HGF:5-scratch:5	0.000	CD73	scratch + HGF + BMMPs:3-scratch + HGF:3	2.312 × 10^−6^
IL-8	scratch + HGF:1-scratch:1	0.013	CD73	scratch + TNFα + BMMPs:5-scratch:5	8.096 × 10^−5^
IL-8	scratch + HGF:5-HGF:5	0.030	CD73	scratch + BMMPs:5-scratch:5	0.000
IL-8	scratch + TNFα + BMMPs:5-scratch:5	0.032	CD73	scratch + TNFα:5-scratch:5	0.001
IL-8	scratch + HGF:1-HGF:1	0.033	CD73	scratch + TNFα:1-TNFα:1	0.002
IL-8	scratch + TNFα + BMMPs:3-scratch:3	0.0425	CD73	scratch + TNFα + BMMPs:1-scratch + BMMPs:1	0.004
IL-8	scratch + TNFα + BMMPs:1-TNFα:1	0.044	CD73	scratch + HGF + BMMPs:1-scratch:1	0.006

**Table 2:** The most statistically significant conditions plus time points for IL-8, FGF-1, and SCF production.

Protein	Condition and Day	Adj. *p*-Value
IL-8	scratch + TNFα + BMMPs:1-control:1	4.37 × 10^−13^
IL-8	scratch+HGF + BMMPs:1-scratch:1	4.37 × 10^−13^
IL-8	scratch + TNFα:3-control:3	4.37 × 10^−13^
IL-8	scratch + TNFα + BMMPs:3-control:3	4.37 × 10^−13^
IL-8	scratch + HGF + BMMPs:3-scratch + HGF:3	0.003
IL-8	scratch + HGF + BMMPs:5-scratch + BMMPs:5	0.000
IL-8	scratch + BMMPs:5-scratch:5	0.000
FGF-1	scratch + HGF + BMMPs:1-scratch + BMMPs:1	4.67 × 10^−13^
FGF-1	scratch + TNFα+BMMPs:1-scratch + BMMPs:1	5.18 × 10^−13^
FGF-1	scratch + BMMPs:1-control:1	8.21 × 10^−11^
FGF-1	scratch + TNFα + BMMPs:5-TNFα:5	0.000
FGF-1	scratch + TNFα + BMMPs:3-TNFα:3	0.001
SCF	scratch + BMMPs:1-control:1	4.37 × 10^−13^
SCF	scratch + HGF + BMMPs:1-control:1	4.37 × 10^−13^
SCF	scratch + HGF + BMMPs:3-scratch + HGF:3	4.37 × 10^−13^
SCF	scratch + TNFα + BMMPs:3-scratch:3	4.37 × 10^−13^
SCF	scratch + HGF + BMMPs:3-HGF:3	4.37 × 10^−13^
SCF	scratch + HGF + BMMPs:5-control:5	4.37 × 10^−13^
SCF	scratch + HGF + BMMPs:5-scratch:5	4.37 × 10^−13^
SCF	scratch + HGF + BMMPs:5-scratch + BMMPs:5	4.37 × 10^−13^
SCF	scratch + TNFα + BMMPs:5-scratch + TNFα:5	4.37 × 10^−13^
SCF	scratch + HGF + BMMPs:3-scratch + BMMPs:3	4.38 × 10^−13^

**Table 3:** List of forward and reverse primes used from qPCR.

Gene	Forward Primer (5′-3′)	Reverse Primer (5′-3′)	Fragment Size
*CD34*	TGAAGCCTAGCCTGTCAC	ATAAGACCTCCAGCTGTGCG	180 bp
*CD45*	GTGTTTCATCAGTACAGACG	GCTGTCATTTCAACCACAAC	191 bp
*CD29*	GGATTCTCCAGAAGGTGGTTTCG	GGAGATGGGAAACTTGGTGGCA	121 bp
*CD105*	GGGGTCAACACCACAGAG	CACATCCTGAGGGTCCTG	261 bp
*CD73*	GGATTCTCCAGAAGGTGGTTTCG	CATCGCTCAGAAAGTGAGG	310 bp
*IL-8*	GAGAGTGATTGAGAGTGGACCAC	AAACTGGGTGCAGAGGGTTGTG	90 bp
*ACTB*	AGTCCTAGCTACTCCGGAGGC	CGGCTATTCTCGCAGCTCAC	113 bp

**Table 4:** Test statistics for Two-Way ANOVA testing for ELISA data.

	Condition	Day	Condition:Day
IL-8	F(8) = 544.06; *p*-value < 2 × 10^−16^	F(2) = 107.29; *p*-value < 2 × 10^−16^	F(16,54) = 21.31; *p*-value < 2 × 10^−16^
FGF-1	F(8) = 21.06; *p*-value = 4.55 × 10^−14^	F(2) = 85.82; *p*-value < 2 × 10^−16^	F(16,54) = 10.86; *p*-value = 1.17 × 10^−11^
SCF	F(8) = 2658.3; *p*-value < 2 × 10^−16^	F(2)= 1682.7; *p*-value < 2 × 10^−16^	F(16.54)= 863.1; *p*-value < 2 × 10^−16^

**Table 5:** Test statistics for Two-Way ANOVA testing for qPCR data.

	Condition	Day	Condition:Day
CD73	F(7) = 219.7; *p*-value < 2 × 10^−16^	F(2) = 752.8; *p*-value < 2 × 10^−16^	F(14,48) = 397.0; *p*-value < 2 × 10^−16^
IL-8	F(7) = 93.248; *p*-value < 2 × 10^−16^	F(2) = 3135.047; *p*-value < 2 × 10^−16^	F(14,48) = 0.769; *p*-value = 0.696

**Figure A4:**
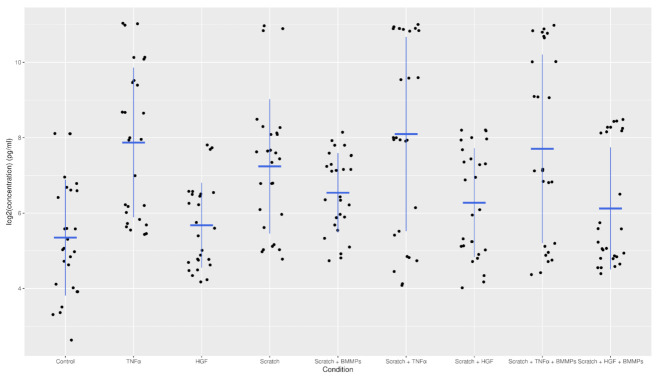
Strip plot of log2 concentration in pg/mL of FGF-1, IL-8, and SCF against the different conditions for the three biological replicates.

## Data Availability

The data is contained within the article or supplementary material.

## Supplementary Materials

The Supplementary Materials are available online at https://www.mdpi.com/article/10.3390/ijms23010078/s1.
